# Supplementary material for: Influence mechanism of Internet use on the physical and mental health of the Chinese elderly—Based on Chinese General Social Survey
Source: PLoS One. 2025 Jan 3;20(1):e0312664. doi: 10.1371/journal.pone.0312664 (PMC11698357; doi:10.1371/journal.pone.0312664)
Supplement: Attachment — Submitted filename: Reviewer Feedback.docx [file pone.0312664.s002.docx]

## General Assessment

The manuscript under review investigates the impact of Internet use on the physical and mental health of elderly individuals, utilizing data from the Chinese General Social Survey and applying binary logistic regression and stepwise regression analyses. The study concludes that Internet use significantly enhances both the physical and mental well-being of elderly people. It identifies physical exercise, social interaction, and learning behavior as partial mediators in this relationship, with learning behavior having the strongest mediating effect.

Overall, while the manuscript is well-written and presents a thorough analysis, it lacks innovation in several areas. The literature review is not sufficiently comprehensive or up-to-date, and the theoretical framework and hypotheses are somewhat rigid and underdeveloped. Although three theories are mentioned, they are not explored in depth. I recommend the authors introduce more innovative models and variable selections, expand the literature review, and conduct heterogeneity analysis. The conclusion section should also provide more targeted policy recommendations.

## Critical Evaluation

1. **Definition and Literature Support**

The manuscript's core concept, "Internet use," is not clearly defined or explained in detail. The literature cited is somewhat outdated and needs to be refreshed with more recent studies. The statement "Based on existing research and reality, Internet use has a complex impact mechanism on the physical and mental health of the elderly" lacks adequate literature support, which needs to be addressed. Moreover, the literature review and hypothesis section contain too few citations, and the discussion of the literature is not sufficiently deep. Given that the study focuses on China, it is crucial to emphasize why examining this issue in the Chinese context is important, which is not adequately reflected in the current draft.

Additionally, there are grammatical issues throughout the manuscript. For instance, the sentence “the impact mechanism of Internet use on the physical and mental health of the elderly in China still needs to be further explored and made up for the lack of existing research” is unclear and poorly structured. Another example is “Will never assign a value of 1, rarely assign a value of 2, sometimes assign a value of 3, frequently assign a value of 4, and very frequently assign a value of 5,” which is not written in a standardized manner.

2. **Methodological and Innovation Concerns**

The methodological approach lacks innovation. The study employs only binary logistic regression and the Mediation Effect Model, without introducing any novel methods. The selection of variables is also limited; for instance, there are no control variables related to the elderly individuals' children, which could be relevant to the analysis. Additionally, while the manuscript examines the impact of Internet use on the physical and mental health of the elderly, a topic already well-covered in the literature, it does not offer any substantial innovations. The mediation effects of physical exercise, social interaction, and learning behavior are common factors in the literature, making this study appear quite ordinary. I recommend that the authors focus on developing more innovative models and consider introducing new variables or methods to enhance the study’s contribution.

3. **Conclusions and Policy recommendations**

The policy recommendations provided in the manuscript are not sufficiently robust. Furthermore, the manuscript does not address the heterogeneity of the elderly population, which could be analyzed using the control variables already included in the study. To strengthen the manuscript, I suggest incorporating heterogeneity analysis and offering more specific and actionable policy recommendations in the conclusion

## Areas for Improvement

To strengthen the manuscript, it is recommended that the authors:

**1. Clarify and Define Key Concepts:** Provide a clear and detailed definition of “Internet use” and ensure that the literature review is up-to-date and comprehensive. Expand the discussion to include more recent studies and provide a stronger theoretical grounding for the hypotheses.

**2. Innovate Methodologically:** Introduce novel methods or models to analyze the data. Consider including additional control variables, such as those related to the elderly individuals’ children, to offer a more comprehensive analysis. Incorporate heterogeneity analysis to better understand the diverse effects of Internet use on different subgroups within the elderly population.

**3. Expand Policy Recommendations:** Provide more targeted and actionable policy recommendations.

4. **Improve Writing Quality:** Address the grammatical issues throughout the manuscript and ensure that the writing adheres to academic standards.
