# Supplementary material for: Influence mechanism of Internet use on the physical and mental health of the Chinese elderly—Based on Chinese General Social Survey
Source: PLoS One. 2025 Jan 3;20(1):e0312664. doi: 10.1371/journal.pone.0312664 (PMC11698357; doi:10.1371/journal.pone.0312664)
Supplement: Attachment — Submitted filename: Response to Reviewers.doc [file pone.0312664.s003.doc]

PONE-D-24-24643

**Response to reviewers**

Dear Editor and Reviewers:

Thank you for taking time out of your busy schedule to review the manuscript! First of all, I would like to express my sincere gratitude. Your professional review comments are very important for improving the academic research quality of this article, helping me identify shortcomings and providing constructive guidance for the revision and improvement of the paper. Thanks again! I have carefully read and analyzed all the feedback from the reviewer and editor, and have made corresponding revisions and improvements throughout the entire article. The modifications in the manuscript have been marked in red (*Revised Manuscript with Track Changes.docx*). Revision notes, point-to-point, are given as follows:

**Review-1: Response to the questions one by one**

Here are the general comments from the reviewer:

*The manuscript under review investigates the impact of Internet use on the physical and mental health of elderly individuals, utilizing data from the Chinese General Social Survey and applying binary logistic regression and stepwise regression analyses. The study concludes that Internet use significantly enhances both the physical and mental well-being of elderly people. It identifies physical exercise, social interaction, and learning behavior as partial mediators in this relationship, with learning behavior having the strongest mediating effect.*

*Overall, while the manuscript is well-written and presents a thorough analysis, it lacks innovation in several areas. The literature review is not sufficiently comprehensive or up-to-date, and the theoretical framework and hypotheses are somewhat rigid and underdeveloped. Although three theories are mentioned, they are not explored in depth. I recommend the authors introduce more innovative models and variable selections, expand the literature review, and conduct heterogeneity analysis. The conclusion section should also provide more targeted policy recommendations.*

**Response:** Thank you for the comments and suggestions from the reviewers. Firstly, I have added the latest literature to support the research content in the literature review section; Secondly, I provided a detailed description of the three supporting theories present in my research content; Again, new control variables were introduced and relevant models were replaced; Finally, heterogeneity analysis was conducted and more targeted policy recommendations were provided. To ensure the reviewers and editors have a clear understanding, the following is a detailed explanation of the revisions.

**Critical Evaluation**

1) *Definition and Literature Support*

*The manuscript's core concept, "Internet use," is not clearly defined or explained in detail. The literature cited is somewhat outdated and needs to be refreshed with more recent studies. The statement "Based on existing research and reality, Internet use has a complex impact mechanism on the physical and mental health of the elderly" lacks adequate literature support, which needs to be addressed. Moreover, the literature review and hypothesis section contain too few citations, and the discussion of the literature is not sufficiently deep. Given that the study focuses on China, it is crucial to emphasize why examining this issue in the Chinese context is important, which is not adequately reflected in the current draft.*

*Additionally, there are grammatical issues throughout the manuscript. For instance, the sentence “the impact mechanism of Internet use on the physical and mental health of the elderly in China still needs to be further explored and made up for the lack of existing research” is unclear and poorly structured. Another example is “Will never assign a value of 1, rarely assign a value of 2, sometimes assign a value of 3, frequently assign a value of 4, and very frequently assign a value of 5,” which is not written in a standardized manner.*

**Response:** I gratefully thanks for the precious time the reviewer spent making constructive remarks. I have provided a clear and detailed definition of “Internet use” and ensure that the literature review is up-to-date and comprehensive (the first paragraph in "2 Literature review"). In addition, I expand the discussion to include recent research and provide a stronger theoretical foundation for the hypothesis(in "2 Literature review"). Mainly reflected in "1 Introduction", "2 Literature review". Finally, regarding the grammatical issues present in the manuscript, such as "the impact mechanism of Internet use on the physical and mental health of the elderly in China still needs to be further explored and made up for the shortcomings of existing research", and other relevant sentences have been refined and revised to ensure that reviewers, editors and readers have a clear understanding of the article.

2) *Methodological and Innovation Concerns*

*The methodological approach lacks innovation. The study employs only binary logistic regression and the Mediation Effect Model, without introducing any novel methods. The selection of variables is also limited; for instance, there are no control variables related to the elderly individuals' children, which could be relevant to the analysis. Additionally, while the manuscript examines the impact of Internet use on the physical and mental health of the elderly, a topic already well-covered in the literature, it does not offer any substantial innovations. The mediation effects of physical exercise, social interaction, and learning behavior are common factors in the literature, making this study appear quite ordinary. I recommend that the authors focus on developing more innovative models and consider introducing new variables or methods to enhance the study’s contribution.*

**Response:** Thank you very much for the valuable opinions of the reviewer and the editor! We have carefully learned to your suggestions and have specifically added the following content. Firstly, I added control variables related to the children of the Chinese elderly to provide a more comprehensive analysis (see "Table 1 Variable definition and description statistics"). Secondly, I introduced a new model to analyze the data to ensure its accuracy (see "3.3 Model settings"); Finally, the heterogeneity test and analysis part is added to better understand the different influence of Internet use on different categories of the Chinese elderly, that improve the innovation of the article, and increase the contribution of the article (see "4.3 The influence of Internet use on the physical and mental health of the Chinese elderly: a heterogeneity analysis based on socio-economic status ").

Supplementary content:

Table 1 Variable definition and description statistics

| Variables | Variable Description | Mean  /percentage | Standard deviation | Variable characteristics | Sample size |
| --- | --- | --- | --- | --- | --- |
| Dependent variable |  |  |  |  |  |
| Physical health | Unhealthy=0, healthy=1 | 0.375 | 0.484 | Categorical scale variables | 2772 |
| Mental Health | Unhealthy=0, healthy=1 | 0.635 | 0.482 | Categorical scale variables | 2772 |
| Core independent variable |  |  |  |  |  |
| Internet use | Not use=0, use=1 | 0.389 | 0.488 | Categorical scale variables | 2772 |
| Replace independent variables |  |  |  |  |  |
| Surf the Internet or not | Not=0, yes=1 | 0.351 | 0.477 | Categorical scale variables | 2772 |
| Control variable  Personal characteristics |  |  |  |  |  |
| Age | 60 to 75 years=0,  75 years and above=1 | 24.6% | 0.431 | Categorical scale variables | 2771 |
| Gender | Female=0, male=1 | 49.1% | 0.500 | Ordered variable | 2772 |
| Marriage | No spouse=0, with spouse=1 | 71.0% | 0.454 | Categorical scale variables | 2772 |
| Registered residence | Rural=0, urban and other=1 | 41.4% | 0.493 | Categorical scale variables | 2772 |
| Education | Uneducated =0, educated=1 | 79.4% | 0.405 | Categorical scale variables | 2757 |
| Social characteristics |  |  |  |  |  |
| Labor Participation | Not participating=0,  participating=1 | 0.263 | 0.440 | Categorical scale variables | 2772 |
| Family characteristics |  |  |  |  |  |
| Economic performance | Medium or low=0, high=1 | 0.083 | 0.276 | Categorical scale variables | 2772 |
| Children | From 0 to 13 | 2.307 | 1.285 | Ordered variable |  |
| Social insurance |  |  |  |  |  |
| Basic medical insurance | Not participating=0,  participating=11 | 93.7% | 0.243 | Categorical scale variables | 2772 |
| Basic pension insurance | Not participating=0,  participating=1 | 80.0% | 0.400 | Categorical scale variables | 2772 |
| Mediating variables |  |  |  |  |  |
| Physical exercise | never=1，several times a year=2，several times a month=3，several times a week=4，every day=5 | 2.724 | 1.785 | Ordered variable | 2772 |
| Social interaction | never=1，rarely=2，sometimes=3，frequently=4，very frequently=5 | 2.541 | 1.254 | Ordered variable | 2772 |
| Learning frequency | never=1，rarely=2，sometimes=3，frequently=4，very frequently=5 | 1.737 | 1.129 | Ordered variable | 2772 |

3) *Conclusions and Policy recommendations*

*The policy recommendations provided in the manuscript are not sufficiently robust. Furthermore, the manuscript does not address the heterogeneity of the elderly population, which could be analyzed using the control variables already included in the study. To strengthen the manuscript, I suggest incorporating heterogeneity analysis and offering more specific and actionable policy recommendations in the conclusion.*

**Response:** Thank you for the friendly comments from the reviewer. Firstly, based on the reviewer's suggestion, I have added heterogeneity testing and analysis of elderly based on their socio-economic status (see "4.3 The influence of Internet use on the physical and mental health of the Chinese elderly: a heterogeneity analysis based on socio-economic status "). Included analysis and conclusions on heterogeneity in both the discussion and conclusion sections to better connect the framework of the article (see "5 Discussion" and "6 Conclusions" ). Secondly, based on research findings such as influence mechanisms and heterogeneity, more specific and actionable policy recommendations have been provided, mainly reflected in the "6 Conclusions" section. Finally, I added the limitations and prospects of this article to lay a foundation for future research. All specific modifications can be found in "Abstract", "2 Literature Review", "4.3 The influence of Internet use on the physical and mental health of the elderly: a heterogeneity analysis based on socio-economic status", "5 Discussion", "6 Conclusion", "Table 6 Heterogeneity Analysis Based on Socio-economic Statussection" and highlight it in red”.

Supplementary content:

**1) Table 5 Heterogeneity Analysis Based on Socioeconomic Status**

Table 5 Heterogeneity Analysis Based on Socioeconomic Status

| **Variables** | **Physical health** | | | | **Mental health** | | | |
| --- | --- | --- | --- | --- | --- | --- | --- | --- |
|  | **Model E1**  **Medium-low** | **Model E2**  **High** | **Model E3**  **Un-educated** | **Model E4**  **Educated** | **Model F1**  **Medium-low** | **Model F2**  **High** | **Model F3**  **Un-educated** | **Model F4**  **Educated** |
| Internet use | 0.218^*^  (0.093) | 0.759^*^ (0.334) | 0.438  (0.245) | 0.239^*^  (0.096) | 0.282^**^  (0.096) | 1.145^**^ (0.436) | 0.335 (0.242) | 0.306^**^ (0.102) |
| Control variable | Controlled | Controlled | Controlled | Controlled | Controlled | Controlled | Controlled | Controlled |
| Observations | 2772 | 2772 | 2772 | 2772 | 2772 | 2772 | 2772 | 2772 |
| Constant | -0.830^***^  (0.212) | 0.424  (1.002) | -0.452  (0.364) | -0.891^***^ (0.244) | -0.469^*^  (0.206) | -0.689  (1.158) | -0.193  (0.338) | -0.485^*^ (0.245) |

Note: * *p*<0.05, ** *p*<0.01, *** *p*<0.001; the standard error is enclosed in parentheses.

**2) "6 Conclusion" and "7 Limitations and prospects"**

**6 Conclusion**

Internet use has a positive influence on the physical and mental health participation of the Chinese elderly. The higher the degree of Internet use, the higher level of the physical and mental health of the elderly. Physical exercise, social interaction and learning frequency are all intermediary mechanisms of Internet use affecting the physical and mental health of the elderly. They will indirectly promote the physical and mental health of the elderly along the path of the Internet use’s influence on their physical and mental health. Among the three factors, learning frequency has the highest influence on their physical and mental health, while social interaction has the lowest influence on their physical and mental health. Internet use has an unequal influence on the physical and mental health of the Chinese elderly, and has a more obvious reinforcement effect on the elderly with high socio-economic status. The conclusion of this paper clarifies the influence of Internet use on the physical and mental health of the Chinese elderly, and provides policy enlightenment for coping with the social challenges by the aging population in China.

Based on the above conclusions, this research proposes the following three suggestions. First, increase the popularity of Internet use among the Chinese elderly. At present, the rapid development of the Internet has facilitated people's real life, but the popularity of Internet use among the Chinese elderly still needs to be improved. The government should strengthen the inclusive construction of Internet-related public services, narrow the gap in Internet penetration between urban and rural areas, improve the accessibility of Internet use for the elderly, and promote the elderly to share network resources. The social level should give full affirmation and support to the Internet use of the elderly, and give necessary guidance to the Internet use of the elderly. And encourage the elderly to actively participate in Internet use and enhance their social adaptability [53].

Second, accelerate the development of Internet application products suitable for the Chinese elderly. Although Internet products mainly focus on young people, they often ignore the needs of the elderly. With the rapid development of information technology, elderly can improve their physical and mental health through exercise, learning, and socializing, but they face difficulties in operating devices, software, and web pages. On the one hand, it is necessary to launch Internet applications suitable for the elderly as soon as possible to provide them with a better use experience. On the other hand, it is necessary to simplify the functions of terminal devices used by the elderly for Internet use and reduce the difficulty of use, so that the elderly can meet their own needs for continuous socialization while enjoying the convenient life brought by the Internet.

Third, provide Internet education for different elderly groups. Implement targeted assistance for the elderly people with poor socio-economic status. By expanding the coverage of education and training, providing guiding videos through television and social home guidance, the elderly can master the use of the Internet and create an Internet learning field for themselves. On the one hand, enabling the elderly to achieve social participation through the Internet provides a good environment, which can effectively promote the frequency of Internet use by the elderly and expand the number of elderly using the Internet. On the other hand, strengthen the awareness of lifelong learning of the elderly, so that the use of the Internet can better play the role of information transmission, interpersonal communication and learning promotion.

**7 Limitations and prospects**

Certainly, this article still has limitations. Firstly, although the breadth and depth of the data used in the study are sufficient, the timeliness is weak; Secondly, some studies suggest that the health status of elderly is influenced by factors such as environment and genetic inheritance. Due to limitations in conditions, this manuscript was unable to fully incorporate these factors into the analysis. Third, given the complexity of the relationship between internet use and health, future research should consider longitudinal analysis to understand better the causal relationships and temporal dynamics. Nonetheless, the research offers valuable evidence for seeking a deeper understanding of the relationship between internet use and health. In the future, with the abundance of survey data, hope more detailed discussions can be conducted in further research.

**Review-2: Response to the questions one by one**

*The study used the CGSS data to analyze the impact of Internet using to the health of elderly people. Overall, the manuscript is well-written and the method is sound. My concerns are as follows.*

First of all, thank you for your meticulous guidance and inclusive suggestions on this article. Your review comments are very important for improving the academic research quality and details of this article, helping us further discover shortcomings. We carefully read and analyzed all the feedback from the reviewer, and made corresponding revisions and improvements throughout the entire document.

(1) *The Materials and Methods section is too long, in particular the “Research hypotheses” subsection. Most of the text should be discussed in the “Literature review” section while keeping the Methods section concise.*

**Response**: Thank you to the reviewer for carefully reading and promptly identifying the issues in the article. Based on the reviewer's suggestion, we have moved the "Research Hypothesis" section to "Literature Review" section for discussion, and further streamlined the content of the Methods section to make the entire Methods section more concise. The specific modifications are highlighted in red in "3 Methods".

(2) *The description on the variables is also unnecessarily long. Most of it is just to repeat the contents in Table 1.*

**Response**: Thank you for the valuable suggestions from the reviewer! The description of variables in the text does indeed overlap with the table. Based on the reviewer's suggestion, I have removed duplicate descriptions while retaining necessary variable descriptions. The variable descriptions are mainly reflected in Table 1 to maintain the simplicity of the methodology section. The specific supplementary content has been highlighted in red in "3.2 Variable measurement".

(3) *Why many variables are called “Dummy variable” in Table 1. They are categorical scale variables.*

**Response**: Thank you to the reviewer for carefully reading and providing professional revision suggestions! I have changed the "dummy variables" to "categorical scale variables". The specific modifications are highlighted in red in Table 1.

(4) *The interpretation of eq (1) in “Model Settings” and the formula for the OR value is wrong.*

**Response**: Thank you very much for the reviewer's detailed suggestions, which helped us point out the issue. I have replaced the "binary logistic regression models" and corrected the relevant description. Considering the length and fluency of the article, unnecessary descriptions unrelated to the main topic have been removed to make the structure and descriptions of the article clearer. If the editors and reviewers feel it is necessary, I can also add it back.

(5) *I would like suggest to use the forest plot of the confidence intervals of the OR values to represent the results instead of Tables.*

**Response**: Thank you very much for the valuable opinions of the reviewers! As shown in Fig 1, I have used the forest plot to display the results of the OR confidence interval, making the article fluent and the effect more aesthetically pleasing.

Note: The horizontal line represents the 95% confidence interval.

Fig 1. Binary Logistic Regression Results of Internet Use Affecting Physical and Mental Health

(6) *The language should be improved.*

**Response:** Thank you for the friendly comments from the reviewer. We deeply apologize for the language issues! To make up for this deficiency, we have once again polished and adjusted the language expression and writing throughout the article, hoping to convey the meaning more smoothly. Finally, I would like to thank the reviewer again for the constructive suggestions, which have helped me continuously improve the academic research quality of this article!

Finally, I also polished and revised the text throughout the entire paper, adjusted titles at all levels that meet the requirements of the journal, removed the highlights of the article, modified the format of the references (all highlighted in red font). To sum up, I would like to express my gratitude to the reviewers and the editors in charge for providing important revision guidance for this article, which has helped me continuously improve the academic research quality of this article. If you have any further suggestions for modification, please do not hesitate to contact me! Once again, thank you very much for your comments and suggestions!

Best wishes to you!

Author: Peng HOU
